# Surgical Treatment of a Rare Case of Extrapelvic Endometriosis in the Rectus Abdominis Muscles With Negative Imaging Findings: A Case Report and Mini Literature Review

**DOI:** 10.7759/cureus.73891

**Published:** 2024-11-18

**Authors:** Efthymia Thanasa, Anna Thanasa, Evangelos Kamaretsos, Gerasimos Kontogeorgis, Ioannis Paraoulakis, Ioannis Thanasas

**Affiliations:** 1 Department of Health Sciences, Medical School, Aristotle University of Thessaloniki, Thessaloniki, GRC; 2 Third Department of Obstetrics and Gynecology, University General Hospital "ATTIKON" Medical School, National and Kapodistrian University of Athens, Athens, GRC; 3 Department of Obstetrics and Gynecology, General Hospital of Trikala, Trikala, GRC

**Keywords:** abdominal wall, case report, endometriosis, histological examination, rectus abdominis muscles, surgical treatment, symptoms

## Abstract

Abdominal wall endometriosis is an uncommon clinical entity. The localization of the disease in the muscles of the abdominal wall is considered extremely rare. Our patient with two cesarean sections in her obstetric history presented to the gynecology outpatient clinic of the General Hospital of Trikala, Trikala, Greece, complaining of intense pain, particularly during menstruation, though no palpable lesions were found in the abdominal wall. The pelvic imaging revealed no abnormalities. Based on the clinical findings, endometriosis of the abdominal wall was suspected. Surgical excision of a flat lesion from the abdominal wall muscles, followed by histological examination of the surgical specimen, confirmed endometriosis of the rectus abdominis muscle. The patient's postoperative course was smooth. Six months after surgery, without additional hormonal suppressive medication, the patient reported complete relief of symptoms. To date, she is regularly followed up at the Gynecology outpatient clinic. The remarkable feature of this case is the surgical treatment of endometriosis in the rectus abdominis muscles, based on the typical clinical findings of the disease. The case emphasizes the rarity of endometriosis in the rectus abdominis muscle, the significant challenges in preoperative diagnosis, and the crucial role of recognizing typical clinical features for early diagnosis and effective treatment of abdominal wall endometriosis.

## Introduction

Endometriosis is a hormone-dependent, chronic, inflammatory, and complex condition characterized by the growth and proliferation of endometrial-like tissue outside the uterine cavity. It is a common yet frequently underdiagnosed condition, causing considerable morbidity from puberty through postmenopause [1]. The incidence of endometriosis is estimated to affect up to 10% of women of reproductive age worldwide [2]. Despite the increasing prevalence of the disease and the great interest of the scientific community in these patients in recent years, the pathogenetic mechanisms of endometriosis remain inadequately understood [3]. Endometriosis is typically located on the outer walls of the uterus, the ovaries, the pelvic peritoneum, and the uterosacral ligaments (intrapelvic endometriosis). In rare cases, the disease may extend throughout the peritoneal cavity up to the diaphragm (extrapelvic endometriosis) [4,5]. Extrapelvic localization of the disease in the gastrointestinal tract is most common in the large intestine and significantly less common in the small intestine [6]. Other uncommon extrapelvic sites include the urinary tract, nervous system, perineum, umbilicus, and abdominal wall [7-10].

Abdominal wall endometriosis is estimated to represent 0.03%-2% of extrapelvic forms of the disease [11]. It most commonly occurs following obstetric or gynecologic surgery involving laparotomy or laparoscopy [12]. Localization within the rectus abdominis muscles (our case) is considered extremely rare [13]. Endometriosis in the abdominal wall muscles was first described by Amato and Levitt in 1984 [14]. The prevalence of rectus abdominis muscle endometriosis, particularly when associated with cesarean section, is reported to range from 0.03% to 0.45% [15]. It is estimated that approximately 25% of these patients have a history of pelvic endometriosis. Symptom onset usually occurs within three months to 10 years after surgery [15]. Our patient had two cesarean sections in her obstetric history. Our patient, who had undergone two cesarean sections, experienced symptom onset of endometriotic foci in the rectus abdominis muscles nine years after her last cesarean section. Notably, there was no history or imaging evidence of pelvic endometriosis in this case.

A distinctive aspect of this case was the decision to proceed with surgical treatment based on clinical diagnosis despite negative pelvic imaging and the rarity of endometriosis in the rectus abdominis. This underscores the considerable diagnostic challenges presented by such cases and highlights the importance of recognizing typical clinical features of abdominal wall endometriosis, which can lead to timely and accurate preoperative diagnosis and intervention.

## Case presentation

A 34-year-old reproductive patient with two cesarean sections in her obstetric history presented to the gynecology outpatient clinic of the General Hospital of Trikala, Trikala, Greece, complaining of lower abdominal pain along the Pfannenstiel incision. Her most recent cesarean section was performed 10 years ago, and the onset of the pain was approximately nine years following this surgery. The pain was continuous and had progressively intensified over the past few months, with the patient reporting that it became even more severe during menstruation. On abdominal examination, no palpable mass was detected in the abdominal wall. The patient reported increased tenderness on deep palpation along the surgical site, particularly near its right end. Ultrasound, computed tomography, and magnetic resonance imaging of the abdomen revealed no abnormal findings from the abdominal wall or pelvic organs. Inflammation markers and tumor markers were within normal ranges (Table 1).

**Table 1:** Inflammatory markers and cancer markers during the patient's preoperative checkup WBC: white blood cells; NEUT: neutrophils; CRP: C reactive protein; CEA: carcinoembryonic antigen; CA125: cancer antigen 125; CA15-3: cancer antigen 15-3; CA15-9: cancer antigen 19-9

Laboratory tests	Preoperative values	Normal laboratory values
WBC	5.68 x 40^3^/mL	4-10.8 x 10^3^/mL
NEUT	62.9%	40%-75%
CRP	0.1 mg/dL	0.5 mg/dL
CEA	2.75 ng/mL	<5 ng/mL
CA125	17.1 U/mL	≤35 U/mL
CA15-3	18.7 U/mL	0.0-31.3 U/mL
CA15-9	14.9 U/mL	0.0-37 U/mL

The patient had a medical history of well-regulated hypothyroidism, for which she was receiving appropriate medication. Her body mass index (BMI) was within the normal range (BMI = 23).

**Figure 1:**
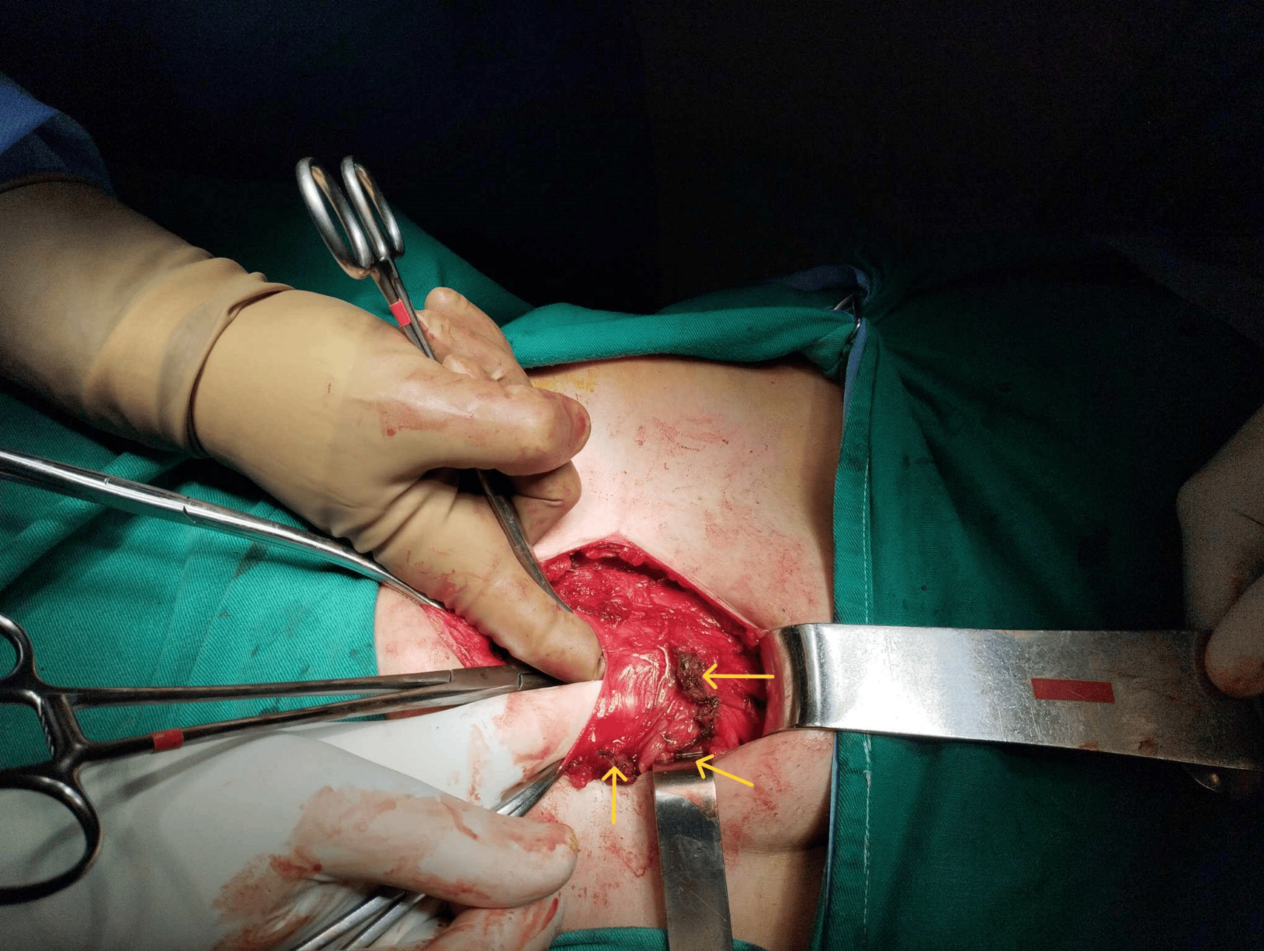
Intraoperative image of rectus abdominis endometriosis. The flat form of the disease is evident bilaterally within the rectus abdominis muscles and especially on the right side (yellow arrows)

Despite the negative pelvic imaging, abdominal wall endometriosis was strongly suspected based on the patient's medical history and clinical findings. A decision was made to surgically investigate the abdominal wall, particularly around the previous surgical sites. No damage to the subcutaneous tissue was observed. However, after dissection and opening of the fascia, a flat mass was identified at the level of the rectus abdominis muscles, located near the pubic symphysis, particularly on the right side. The mass was found to infiltrate the muscle wall (Figure 1).

**Figure 2:**
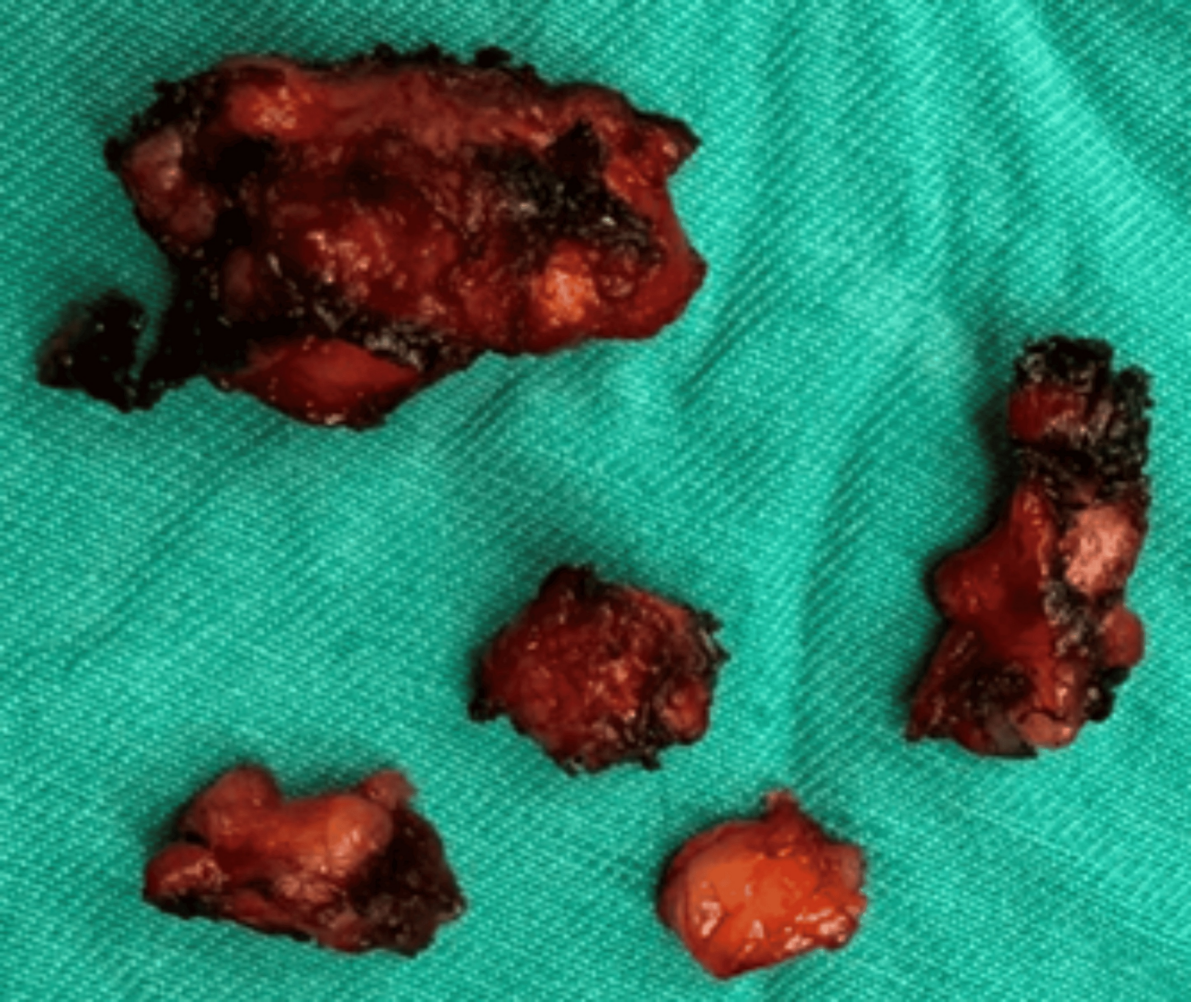
Surgical specimen of rectal abdominal muscle endometriosis. Sections of fibroadipose and striated muscle tissue excised from the rectus abdominis muscles are shown

Wide segmental excision of the lesion was performed, and macroscopic examination revealed sections of fibroadipose and striated muscle tissue with foci of endometriosis (Figure 2).

**Figure 3:**
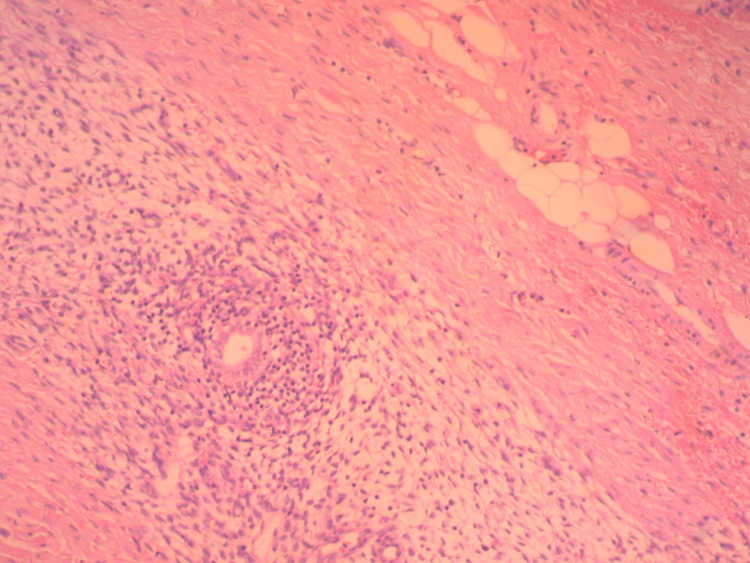
Histological image of rectal abdominal muscle endometriosis. The presence of dilated endometrial glands and endometrial stroma with hemorrhagic infiltration is clearly depicted (hematoxylin and eosin stain; magnification 10×)

Microscopic examination of the surgical specimen detected the presence of dilated endometrial glands and endometrial stroma, which confirmed the diagnosis of endometriosis of the rectus abdominis muscles (Figure 3).

After evaluating the abdominal wall, the surgical team determined that mesh placement was necessary. Our patient was discharged on the second postoperative day. No additional hormonal suppressive therapy was recommended, as it was considered that complete resection of the endometriotic lesion had been achieved. Six months after surgery, our patient reported full relief of preoperative symptoms. To date, she has been under regular follow-up (every six months) at the gynecology outpatient clinic.

## Discussion

Diagnosing endometriosis of the rectus abdominis muscles preoperatively is challenging. However, the presence of a localized, palpable mass near the surgical site of prior obstetric or gynecological surgery involving the uterine cavity is a significant risk factor for abdominal wall endometriosis. A key clinical indicator of anterior abdominal wall endometriosis is cyclic pain that intensifies progressively during menstruation [16]. In some cases, however, this typical cyclic pain may be absent, and the diagnosis may only occur incidentally during surgery for another condition or may present as mild tenderness on palpation of the suprapubic area along the surgical site [17]. In exceptionally rare cases, the pain may be acute and extremely severe due to the formation of an endometrioma at the rectus abdominis muscles [18]. In our patient, the history of previous cesarean sections, combined with the typical persistent and intense cyclic pain localized to the surgical site, strongly indicated the likelihood of abdominal wall endometriosis. Despite the absence of imaging findings of endometriosis in the abdominal wall and pelvis, surgical treatment of the patient was decided based exclusively on the typical clinical features of the disease.

While the diagnostic value of typical clinical features in abdominal wall endometriosis is well established, modern imaging modalities, such as ultrasound, computed tomography, and magnetic resonance imaging, play a crucial role in achieving early and accurate preoperative diagnoses. Transabdominal and Doppler ultrasound imaging techniques of the abdominal wall are particularly valuable for detecting endometriosis in the rectus abdominis muscles. Ultrasound findings can vary widely, presenting as a solid, cystic, or mixed mass with solid and cystic components, which may appear hypoechoic or hyperechoic and often display high vascularity on Doppler imaging [18,19]. Computed tomography and magnetic resonance imaging provide valuable diagnostic information, especially for detecting small, flat masses, by identifying the precise location and delineating the size of the lesion in the abdominal wall. At the same time, it is considered that they can significantly assist in making decisions for the appropriate planning of an early therapeutic approach to the disease [16]. However, in our patient, transabdominal ultrasound, computed tomography, and magnetic resonance imaging could not reveal findings on the abdominal wall indicative of endometriotic lesions and establish the diagnosis of endometriosis in the rectus abdominis muscle. Most likely, this is attributed to the presence of flat and diffuse lesions within the abdominal muscle wall. Consequently, the diagnosis and the decision to surgically investigate the disease were based exclusively on the clinical findings.

Surgical treatment remains the primary treatment option, especially for large extrapelvic endometriomas and for nonpalpable or intermittently palpable lesions in the abdominal wall [20]. Wide local excision of the lesion with clear margins (5-10 mm of surrounding normal tissue) is the treatment of choice, ensuring the histological diagnosis of endometriosis of the rectus abdominis muscle and minimizing recurrence risk. Surgical excision of the lesion should be performed with great care to prevent tissue damage and potential reimplantation of microscopic remnants of endometriosis disease in the adjacent tissues [21]. In cases of large endometriomas or extensive flat endometriotic lesions, the resection of which results in a significant deficit in the rectus abdominis muscles, mesh placement is necessary. Mesh placement is especially beneficial for replenishing the tissue loss caused by resection of the endometriotic mass [12,13]. In our patient, a team of specialized surgeons positioned mesh at the level of the rectus abdominis muscles to reinforce the area. Additionally, thorough irrigation of the surgical wound with normal saline at the end of the procedure and changing surgical gloves before suturing the abdominal wall are considered good surgical practices. These measures, although not conclusively proven, may help reduce the risk of abdominal wall endometriosis recurrence [11].

Hormonal suppressive therapy can help manage symptoms and delay the progression of endometriosis in symptomatic patients who do not wish to achieve pregnancy immediately. The first-line treatment typically includes combined oral contraceptives and progestogens. If these fail to provide adequate symptom control, second-line options such as gonadotropin-releasing hormone agonists and antagonists may be considered [22]. Pharmacological therapy, not combined with surgery, is thought to provide temporary relief from symptoms but is not able to effectively address the underlying disease. Additionally, studies indicate a significant risk of recurrence following the discontinuation of medication [23]. Postoperative hormonal suppressive therapy may be beneficial for patients where there is suspicion of incomplete excision of an endometriotic mass from the abdominal wall on nonclear margins, as it can help reduce the risk of recurrence in cases [16,24]. In our patient, no additional hormonal suppressive therapy was recommended postoperatively, as it was considered that complete excision of the endometriotic foci from the rectus abdominis muscles was performed.

The prognosis of rectus abdominis endometriosis is generally favorable, with a recurrence rate of up to 4.3%. Malignant transformation is rare (0.3%-1%) [24]. In patients where there is a strong suspicion of malignant transformation of the endometriotic lesion, the therapeutic approach should prioritize prompt surgical intervention, potentially followed by adjuvant therapy as appropriate [25].

## Conclusions

Abdominal wall endometriosis located in the rectus abdominis muscles is an extremely rare nosological entity. In symptomatic patients with relevant risk factors and typical clinical signs of the disease, negative pelvic imaging should not rule out the possibility of abdominal wall endometriosis. Misinterpretation or underestimation of clinical symptoms may delay accurate preoperative diagnosis and the timely application of effective treatment, potentially impacting patient quality of life and leading to disease progression.
